# Kinematic strategies for obstacle-crossing in older adults with mild cognitive impairment

**DOI:** 10.3389/fnagi.2022.950411

**Published:** 2022-12-13

**Authors:** Shiuan-Huei Lu, Yi-Chun Kuan, Kuan-Wen Wu, Hsuan-Yu Lu, Yu-Lin Tsai, Hsiang-Ho Chen, Tung-Wu Lu

**Affiliations:** ^1^Department of Biomedical Engineering, National Taiwan University, Taipei City, Taiwan; ^2^Taipei Neuroscience Institute, Taipei Medical University, Taipei City, Taiwan; ^3^Dementia Center and Department of Neurology, Shuang Ho Hospital, Taipei Medical University, New Taipei City, Taiwan; ^4^Department of Neurology, School of Medicine, College of Medicine, Taipei Medical University, Taipei City, Taiwan; ^5^Department of Orthopaedic Surgery, National Taiwan University Hospital, University, Taipei City, Taiwan; ^6^School of Biomedical Engineering, Taipei Medical University, Taipei City, Taiwan; ^7^Department of Biomedical Engineering and Center for Biomedical Engineering, Chang Gung University, Taoyuan City, Taiwan; ^8^Department of Orthopaedic Surgery, School of Medicine, National Taiwan University, Taipei City, Taiwan

**Keywords:** mild cognitive impairment, kinematics strategies, balance control strategies, obstacle-crossing, fall risk

## Abstract

**Introduction:**

Mild cognitive impairment (MCI) is considered a transitional stage between soundness of mind and dementia, often involving problems with memory, which may lead to abnormal postural control and altered end-point control when dealing with neuromechanical challenges during obstacle-crossing. The study aimed to identify the end-point control and angular kinematics of the pelvis-leg apparatus while crossing obstacles for both leading and trailing limbs.

**Methods:**

12 patients with MCI (age: 66.7 ± 4.2 y/o; height: 161.3 ± 7.3 cm; mass: 62.0 ± 13.6 kg) and 12 healthy adults (age: 67.7 ± 2.9 y/o; height: 159.3 ± 6.1 cm; mass: 61.2 ± 12.0 kg) each walked and crossed obstacles of three different heights (10, 20, and 30% of leg length). Angular motions of the pelvis and lower limbs and toe-obstacle clearances during leading- and trailing-limb crossings were calculated. Two-way analyses of variance were used to study between-subject (group) and within-subject (obstacle height) effects on the variables. Whenever a height effect was found, a polynomial test was used to determine the trend. A significance level of α = 0.05 was set for all tests.

**Results:**

Patients with MCI significantly increased pelvic anterior tilt, hip abduction, and knee adduction in the swing limb during leading-limb crossing when compared to controls (*p* < 0.05). During trailing-limb crossing, the MCI group showed significantly decreased pelvic posterior tilt, as well as ankle dorsiflexion in the trailing swing limb (*p* < 0.05).

**Conclusion:**

Patients with MCI adopt altered kinematic strategies for successful obstacle-crossing. The patients were able to maintain normal leading and trailing toe-obstacle clearances for all tested obstacle heights with a specific kinematic strategy, namely increased pelvic anterior tilt, swing hip abduction, and knee adduction during leading-limb crossing, and decreased pelvic posterior tilt and swing ankle dorsiflexion during trailing-limb crossing. The current results suggest that regular monitoring of obstacle-crossing kinematics for reduced toe-obstacle clearance or any signs of changes in crossing strategy may be helpful for early detection of compromised obstacle-crossing ability in patients with single-domain amnestic MCI. Further studies using a motor/cognitive dual-task approach on the kinematic strategies adopted by multiple-domain MCI will be needed for a complete picture of the functional adaptations in such a patient group.

## Introduction

Mild cognitive impairment (MCI) is considered a transitional stage between soundness of mind and dementia (Montero-Odasso et al., 2012). Older people with MCI have an incidence of 12–15% for developing dementia compared to 1–2% in healthy peers (Petersen et al., 1999; Roberts and Knopman, 2013). Mild cognitive impairment is categorized into amnestic (aMCI) and non-amnestic MCI (naMCI) based on whether memory is impaired (Hughes et al., 2011). Amnestic MCI is the more common type with higher progression rates toward dementia than naMCI, while naMCI presents an inconsistent association with subsequent conversion to dementia (Grundman et al., 2004; Yaffe et al., 2006; Petersen, 2011). Cognitive decline in MCI leads to problems with memory, executive function, or attention, affecting instrumental activities of daily living (Saunders and Summers, 2011; Jekel et al., 2015). Depending on the number of impaired cognitive domains, each type of MCI can be further categorized into single-domain (impaired memory only) and multiple-domain subtypes (Roberts and Knopman, 2013), single-domain aMCI being the most prevalent (Brodaty et al., 2013; Roberts and Knopman, 2013). Previous clinical studies have reported abnormal postural control, balance disorders during gait, and increased risk of falls in patients with MCI (Shin et al., 2011; Nascimbeni et al., 2015; Lipardo et al., 2017), especially during obstacle negotiation (Robinovitch et al., 2013). Obstacle-crossing requires utilization of motor planning and attentional resources (Clark et al., 2014), adjusted by higher functions of the central nervous system (CNS; Haefeli et al., 2011). Identifying the control strategies adopted by MCI patients during walking and crossing obstacles will be helpful for developing strategies for reducing fall risks in such patient populations.

During obstacle-crossing, the motions of the individual joints are controlled to maintain dynamic body balance while allowing the swing limb to cross the obstacle with sufficient foot-obstacle clearance (Chen and Lu, 2006). Since the human pelvis-leg apparatus is a multi-link system, a change in the angle of a joint may lead to angular changes at other joints, which together determine the end-point position of the swing limb. Such inter-joint and joint-to-end-point kinematic relationships can vary among subject groups and motor tasks, reflecting the neuromusculoskeletal control of the person. Any alterations of the joint kinematics as a result of injury or pathology of the neuromusculoskeletal system will affect the inter-joint and joint-to-end-point kinematic coordination for a successful obstacle-crossing. Through synthesizing the kinematic changes of individual joints and end-points, the kinematic strategy of obstacle-crossing could be identified. This multi-link system approach has been successfully used in revealing the kinematic strategies of obstacle-crossing in various populations (Hsu et al., 2016; Chien and Lu, 2017; Kuo et al., 2017; Wu et al., 2019b). For example, older people with type II diabetes mellitus have been shown to cross obstacles with reduced swing hip adduction and swing knee flexion associated with reduced toe-obstacle clearance, which is regarded as the risk of tripping over obstacles (Hsu et al., 2016). Therefore, data of joint coordination changes enable the kinematic control strategies during obstacle-crossing, as well as the risk factors for falling, to be identified (Sparrow et al., 1996; Pieruccini-Faria et al., 2014).

The risk of falling increases linearly with the number of risk factors. Thus, the accumulated effects of disease and obstacle-crossing may predispose an older person to falling (Tinetti et al., 1988). Increasing obstacle height may also present an increased risk and has been the subject of extensive research (Lu et al., 2006; Chien and Lu, 2017). With increasing obstacle-height, the joint angles of the swing limb are increased (Chou and Draganich, 1997) but those of the stance limb reduced (Hsu et al., 2016). However, it has been shown *via* a multi-objective optimal control technique that the overall control strategy for obstacle-crossing in young adults is independent of obstacle height, suggesting a CNS-maintained motor program (Lu et al., 2012). A recent study showed that this centrally maintained control strategy is altered with aging (Kuo et al., 2021). Normal aging-related cognitive decline has also been shown to affect toe-obstacle clearance (Sakurai et al., 2021). For patients with MCI, previous gait analysis studies have reported deviations, including temporal–spatial parameters such as reduced speed and stride length but greater stride width (Fuentes-Abolafio et al., 2021), and joint kinematics such as increased peak extension and ranges of motion at the knee (Zhong et al., 2021). With impaired cognitive functions and abnormal gait performance (Verghese et al., 2008), older people with MCI may have greater difficulty than their healthy peers in dealing with the neuromechanical challenges during obstacle-crossing. Deficits in higher-order cognitive functions have also been shown to limit obstacle negotiation capabilities in MCI, showing reduced crossing speeds with increased step length variabilities during crossing, and risk of falls (Pieruccini-Faria et al., 2019). While no study has reported whether MCI might affect the control strategies during obstacle-crossing, as a central degenerative disease, it was possible that the CNS-maintained kinematic strategy for obstacle-crossing found in healthy people might be affected. Identifying the altered kinematic changes in individual joints and end-points in older people with MCI when crossing obstacles of different heights will be helpful for a better understanding of kinematic strategies required or developed to overcome the neuromechanical challenges.

The purpose of this study was to quantify the kinematic changes in individual joints and end-points of the pelvis-leg apparatus in older adults with single-domain aMCI during obstacle-crossing as compared to healthy controls. The kinematic strategy adopted by the aMCI group was also identified by synthesizing the kinematic changes for different obstacle heights. It was hypothesized that older adults with aMCI would adopt a specific kinematic strategy with altered joint kinematics and end-point positions for obstacle-crossing, and that such strategy would not be affected by the obstacle height.

## Materials and methods

### Subjects

This study was approved by the Taipei Medical University Joint Institutional Review Board (IRB Permit number: N201903100). All the experiments and procedures were carried out following the Ethical Principles for Medical Research Involving Human Subjects (World Medical Association Declaration of Helsinki). Twelve patients with single-domain aMCI (MCI group; male/female: 7/5; age: 66.7 ± 4.2 years; height: 161.3 ± 7.3 cm; mass: 62.0 ± 13.6 kg) and twelve healthy adults (Control group; male/female: 4/8; age: 67.7 ± 2.9 years; height: 159.3 ± 6.1 cm; mass: 61.2 ± 12.0 kg) were recruited from the university hospital between October 2019 and January 2021. The MCI and Control groups were matched by age, sex, years of education, body height, and body mass (Table 1). Each subject gave informed written consent as approved by the IRB. The diagnosis of aMCI was made by a senior neurologist (YCK) based on the following criteria: (1) presence of subjective cognitive complaints; (2) objective cognitive impairments (defined as 1.5 standard deviations (SD) below the age- and education-corrected normative means) in memory domain, based on neuropsychological assessment (Table 1); (3) Clinical Dementia Rating (CDR) global scale score of 0.5; (4) preserved activities of daily living confirmed by clinician’s interviews; and (5) absence of dementia determined by the Diagnostic and Statistical Manual of Mental Disorders Fourth Edition, Text Revision (DSM-IV-TR) criteria (Petersen, 2011). A participant would be excluded if he/she was unable to walk independently or communicate to complete the clinician’s interview, had severe uncorrected visual or auditory disorders, was functionally dependent, had a central nervous system lesion or severe neuromusculoskeletal disorders, or had undergone surgery of the lower limbs that would affect their gait performance. None of the subjects wore bifocals during daily living or during the gait experiment. An *a priori* power analysis based on pilot results using GPOWER (Erdfelder et al., 1996) determined that a projected sample size of four subjects for each group would be needed with a power of 0.8 at a significance level of 0.05. Thus, 12 subjects for each group were more than adequate for the main objectives of the current study.

**Table 1:** Means (standard deviations) of the demographic characteristic and neuropsychological assessment results for patients with mild cognitive impairment (MCI, *n* = 12) and healthy controls (Control, *n* = 12).

	MCI	Control	*p*-value
**Demographic data**			
Age (years)	66.7 (4.2)	67.7 (2.9)	0.506
Body height (cm)	161.3 (7.3)	159.3 (6.1)	0.454
Body mass (kg)	62.0 (13.6)	61.2 (12.0)	0.874
Sex, number of females/males	5/7	8/4	0.237
Education level (years)	12.5 (3.2)	14.8 (3.7)	0.112
**Neuropsychological assessment**			
CASI	87.0 (7.1)	95.7 (3.8)	0.011*
WSLT recall	3.2 (4.0)	51.5 (29.6)	<0.001*
WSLT recognition	24.5 (25.4)	71.3 (32.9)	0.001*
VF (%)	65.8 (27.1)	92.9 (5.5)	0.005*
WAIS digit span (%)	53.9 (30.5)	78.7 (15.1)	0.023*

*P*-values for between-group comparisons using an independent *t*-test are also given. CASI, cognitive abilities screening instrument; WSLT, word sequence learning Test; VF, verbal fluency test; WAIS, Wechsler Adult Intelligence Scale; *indicated a significant difference with α = 0.05.

### Neuropsychological assessment

Global cognition was assessed using the Cognitive Abilities Screening Instrument (CASI) (Teng et al., 1994), including attention, memory, language abilities, visual construction, list-generating fluency, abstraction, and judgment, giving a total score ranging from 0 to 100. The Word Sequence Learning Test for recall (WSLT Recall) and recognition score (WSLT Recognition) was used to assess memory. A verbal fluency (VF) test was used to assess executive functions and semantic memory (Nutter-Upham et al., 2008). A Digit Span subtest of the Wechsler Adult Intelligence Scale (WAIS Digit Span) was used to assess attention and working memory (Webber and Soble, 2018). The raw scores of WSLT Recall, WSLT Recognition, VF, and WAIS Digit Span were converted to normative data by different age and education ranges (Cauthen, 1978).

### Experiment protocol

Each subject walked at their preferred speed on a 10-m walkway and crossed a tube-like obstacle placed horizontally across a height-adjustable frame with their natural patterns (Lu et al., 2006; Huang et al., 2008). Three obstacle heights (i.e., 10%, 20% and 30% of the subject’s leg length) were included in the experimental trials. Thirty-nine infrared retroreflective markers placed on anatomical landmarks commonly used in human motion analysis were used to track the motions of the body segments, namely anterior superior iliac spines (ASISs), posterior superior iliac spines (PSISs), greater trochanters, mid-thighs, medial and lateral epicondyles, heads of fibulae, tibial tuberosities, medial and lateral malleoli, navicular tuberosities, fifth metatarsal bases, big toes and heels, and mandibular condylar processes, acromion processes, spinous processes of the seventh cervical vertebra (C7), medial and lateral humeral epicondyles, and ulnar styloids (Hong et al., 2015; Wu et al., 2021). Three-dimensional marker trajectories were measured at 200 Hz using an 8-camera motion analysis system (Vicon MX T-40, OMG, United Kingdom) and low-pass filtered using a fourth-order Butterworth filter with a cutoff frequency of 5 Hz before subsequent kinematic analysis (Chien et al., 2013). The ground reaction forces (GRF) were measured at 1200 Hz using three force plates (OR6-7, AMTI, United States) placed on either side of the obstacle in the middle of the walkway and used to determine the toe-offs and heel-strikes during the crossing cycle (Ghoussayni et al., 2004). The starting position of the subject of the trials was adjusted by the examiner so that the subject would step on the force plates naturally without looking at the forceplates. Data for six complete crossing cycles, three for each limb leading, were obtained for each subject in the MCI and Control groups. For the three obstacle-crossing heights, a counterbalanced measures design was used, while the sequence of the obstacle condition was decided by a random number table.

### Crossing speed and end-point parameters

Crossing speed was calculated as the distance traveled by mid-anterior superior iliac spines in the walking direction divided by the time spent from leading toe-off immediately before crossing to trailing heel-strike immediately after crossing. Toe-obstacle clearance for both the leading and trailing limb was calculated as the vertical distance between the toe marker of the swing limb and the obstacle when the swing toe was directly above the obstacle. The trailing toe-obstacle distance was defined as the horizontal distance between the obstacle and the toe marker of the trailing limb during stance immediately before stepping over the obstacle. The leading heel-obstacle distance was defined as the horizontal distance between the obstacle and the heel marker of the leading limb during stance immediately after stepping over the obstacle (Wu et al., 2019b).

### Joint kinematic variables

Each body segment was embedded with an orthogonal coordinate system with the positive x-axis directed anteriorly, the positive y-axis superiorly, and the positive z-axis to the right in accordance with ISB recommendations (Wu and Cavanagh, 1995). The angular motions of the pelvis were described relative to the laboratory coordinate system with the leading limb as the reference limb. Pelvic upward list indicates that the contralateral hip is higher than the ipsilateral hip while ipsilateral rotation indicates that the ipsilateral hip is anterior to the contralateral hip (Wu et al., 2019a). A Cardanic rotation sequence of z-x-y was used to calculate the rotational movements of each lower limb joint (Grood and Suntay, 1983). Effects of soft tissue artifacts of the pelvis-leg apparatus were reduced using a global optimization method that minimized the weighted sum of squared distances between measured and calculated marker positions with joint constraints (Lu and O’connor, 1999). Values of the calculated angular motions when the leading and trailing toes were above the obstacle were extracted for subsequent statistical analysis (Chen and Lu, 2006).

### Statistical analysis

For statistical comparisons between MCI and Control, independent *t*-tests were used for the demographic data and neuropsychological assessment scores, while a two-way mixed-design analysis of variance (ANOVA) was used for the crossing speed, end-point parameters, and all the calculated kinematic variables with one between-subject factor (group) and one within-subject factor (obstacle height). For all the statistical analyses, data from each calculated variable were averaged across crossing cycles for each subject, and data from both sides were further averaged for the two groups. All the calculated variables were determined to be normally distributed by a Shapiro–Wilk test and the homogeneity of variance across groups was confirmed by the Levene’s test. Whenever an obstacle height effect was found, a *post hoc* analysis was performed using a polynomial test to determine the trend. A significance level of α = 0.05 was set for all test conditions. All statistical analyses were performed using SPSS version 20 (SPSS Inc., Chicago, IL, United States).

The multi-link system approach (Hsu et al., 2016; Chien and Lu, 2017; Wu et al., 2019b) was used to synthesize the significant kinematic changes of individual joints and end-points to identify the kinematic strategies of obstacle-crossing in MCI compared to the Control group. Computer simulations using subject-specific multi-link system models were performed to identify the effects of the significant change of an individual joint on the end-point position at instances when the leading and trailing toe was above the obstacle using the model of a typical subject (with a stature closest to the mean of both groups). At each instance, the posture of the model subject was first defined by the mean joint positions of the Control group. With the stance foot fixed to the ground, the joints with significant deviations from the Control were then rotated one at a time according to the mean angular change of the MCI group, while keeping the angles of the other joints fixed and the segments of the stance limb and the segments of the swing limb proximal to the current joint stationary. For each joint rotation, the toe-obstacle clearance was obtained to reveal the effects of the significant angular changes at individual joints on the end-point positions. The simulation results for each joint were then presented using stick figures of the model subject.

## Results

**Figure 1:**
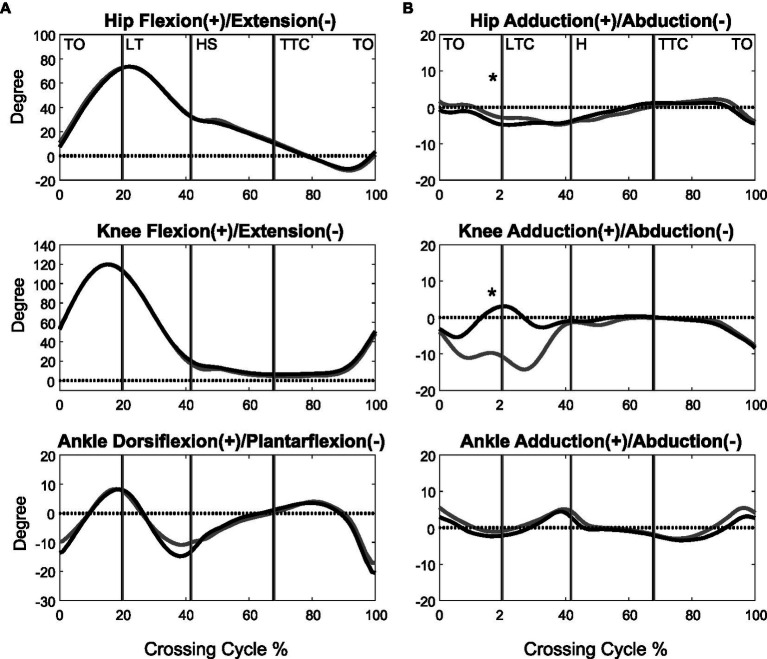
The mean curves of the angles of the hip, knee, and ankle joints of the leading limb in the sagittal **(A)** and frontal plane **(B)** for the MCI (black) and control (grey) groups when crossing obstacles of 30% of leg length. (TO, toe-off of the leading limb; LTC, leading toe above the obstacle; HS, heel-strike of the leading limb; TTC, trailing toe above the obstacle; *significant group effects for all obstacle heights, *p* < 0.05).

**Figure 2:**
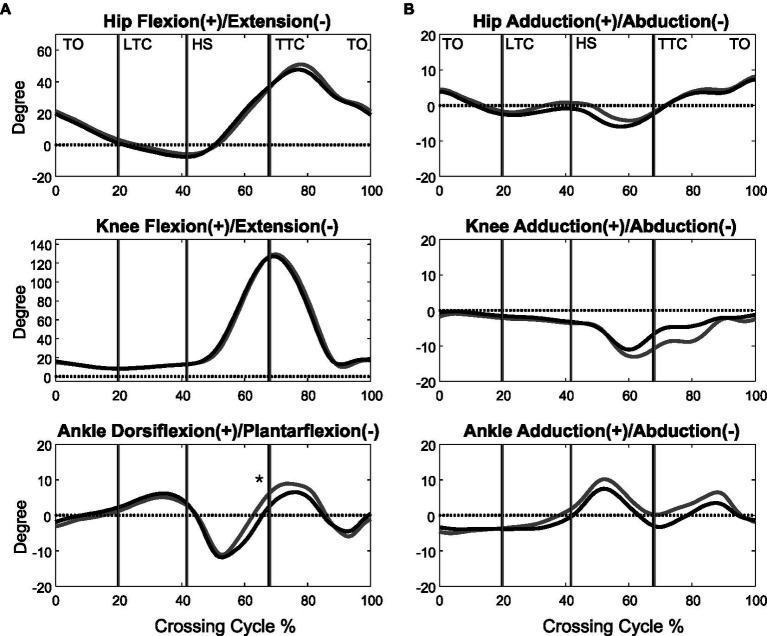
The mean curves of the angles of the hip, knee, and ankle joints of the trailing limb in the sagittal **(A)** and frontal plane **(B)** in the MCI (black) and control (grey) groups when crossing obstacles of 30% of leg length. (TO, toe-off of the leading limb; LTC, leading toe above the obstacle; HS, heel-strike of the leading limb; TTC, trailing toe above the obstacle; *significant main group effect, *p* < 0.05).

Compared to Control, the MCI group showed significantly worse performance in CASI, WSLT Recall, WSLT Recognition, the VF test, and WAIS Digit Span (Table 1). During obstacle-crossing, there were no significant between-group differences in crossing speeds, leading toe-obstacle clearance, trailing toe-obstacle clearance, leading heel-obstacle distance, and trailing toe-obstacle distance (Table 2). The two groups showed similar patterns in the pelvic and lower limb joint motions but with quantitative differences in some kinematic components, primarily in the frontal plane during leading-limb crossing (Figure 1) and in the sagittal plane during trailing-limb crossing (Figure 2).

**Table 2:** Means (standard deviations) of the crossing speeds and end-point parameters for patients with mild cognitive impairment (MCI) and healthy controls (Control) when crossing obstacles of heights of 10, 20, and 30% of the subject’s leg length (LL).

Variables	Obstacle height (% LL)	MCI	Control	P_G_	P_H_
Crossing speed (mm/s)	10	896.5 (146.7)	931.6 (101.4)	0.477	<0.001**↓**
	20	798.7 (124.4)	831.1 (90.4)		
	30	752.0 (128.9)	783.5 (106.8)		
Leading toe-obstacle clearance (% LL)	10	18.7 (4.9)	18.6 (5.2)	0.787	0.308
	20	19.9 (3.8)	19.5 (5.1)		
	30	19.4 (5.0)	21.4 (4.3)		
Trailing toe-obstacle clearance (% LL)	10	16.9 (7.2)	18.0 (10.2)	0.416	0.025↑
	20	16.9 (8.2)	20.0 (10.6)		
	30	18.5 (9.4)	23.0 (8.3)		
Leading heel-obstacle distance (mm)	10	157.3 (54.9)	161.4 (41.9)	0.972	0.531
	20	155.2 (40.8)	149.8 (38.5)		
	30	151.1 (30.8)	150.9 (40.4)		
Trailing toe-obstacle distance (mm)	10	177.7 (23.2)	178.8 (26.4)	0.263	0.449
	20	173.5 (24.1)	185.8 (28.8)		
	30	176.4 (16.0)	191.9 (27.8)		

*P*-values for main group and height effects are given as no interactions were found. P_G_, MCI vs. Control; P_H_, value of *p* for obstacle height; *indicates a significant group effect (P_G_ < 0.05); ↑ indicates a linearly increasing trend and ↓ indicates a linearly decreasing trend (P_H_ < 0.05).

**Figure 3:**
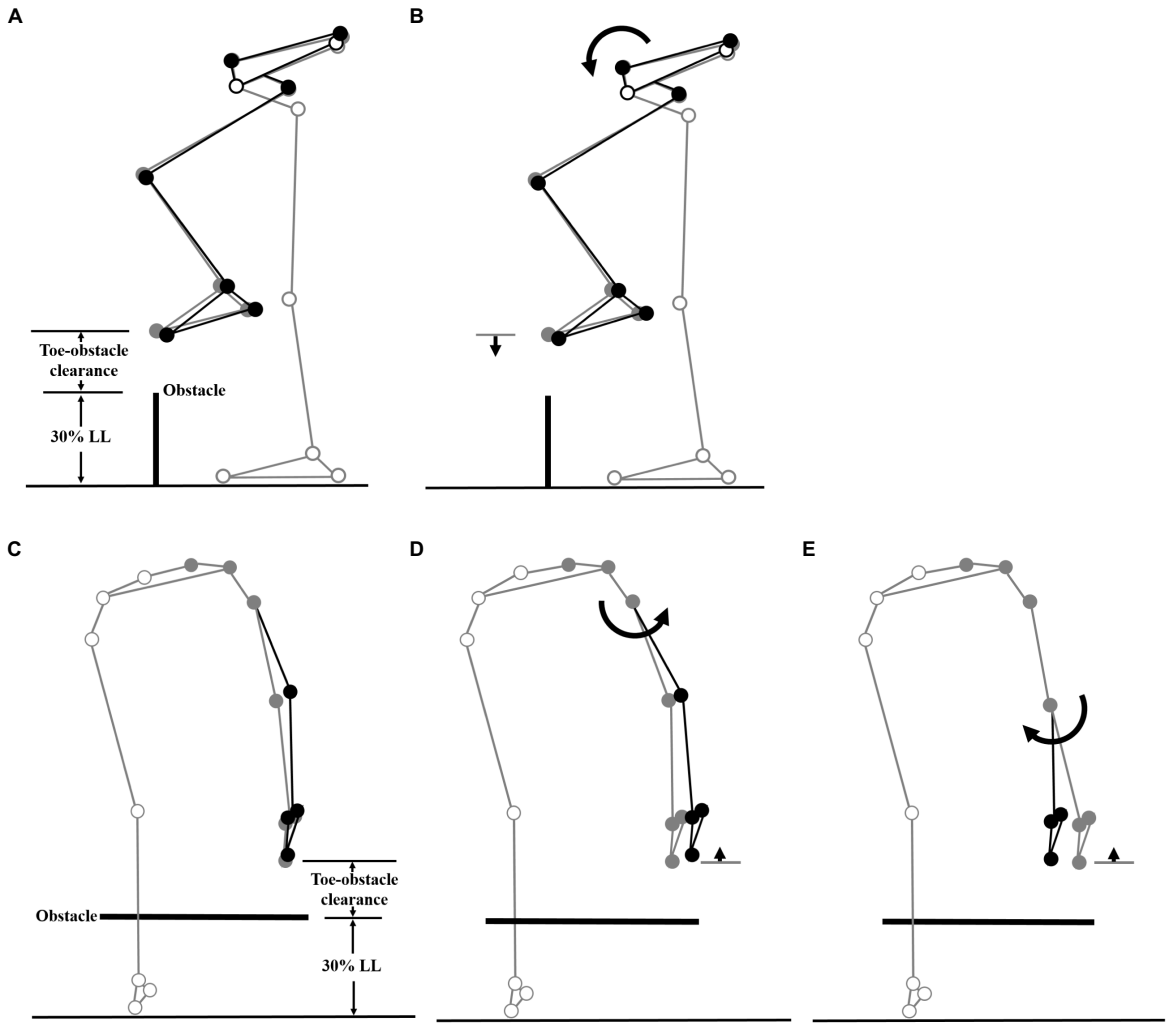
Effects of the observed significant angular changes at individual joints on the leading toe-obstacle clearance in the MCI group (black stick figure) compared to Control (**A** and **C**, grey stick figure) when the leading toe was above an obstacle of 30% LL in height. The stick model was drawn using marker positions of a typical subject from each group. The segments with solid grey circles are joints of the reference limb. With the stance foot fixed to the ground, only one joint was rotated at a time according to the mean angular change reported in Tables 3, 4, while keeping the angles of the other joints fixed, and the segments of the stance limb and the segments of the swing limb distal to the current joint stationary. The MCI group showed increased pelvic anterior tilt **(B)**, and increased hip abduction **(D)** and knee adduction **(E)** in the swing limb. As indicated by the black stick figure, while **(D,E)** tended to increase the leading toe-obstacle clearance, **(B)** gave the opposite effect, leading to the observed normal toe-obstacle clearance. Note that the stick figures were drawn based on the statistical results of the averaged values of both limbs reported in Tables 3, 4.

**Figure 4:**
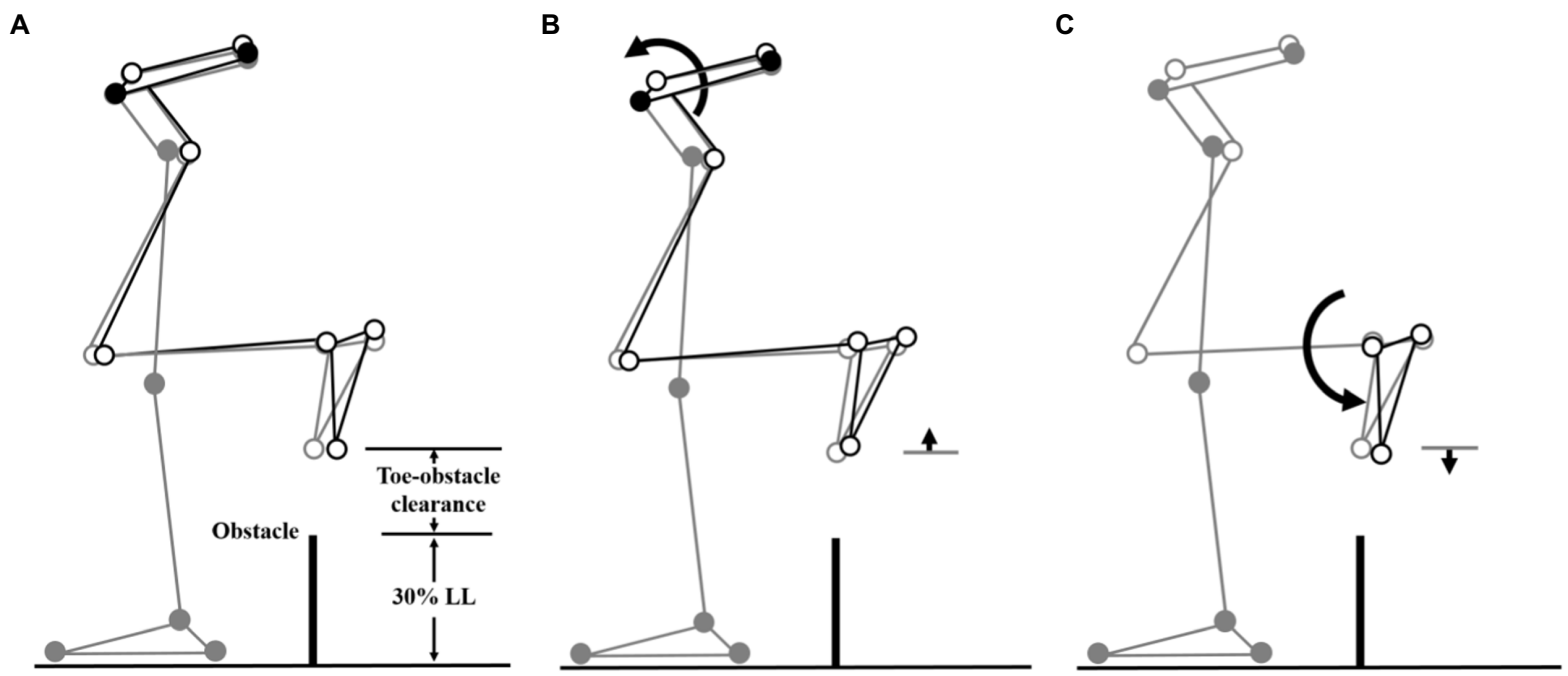
Effects of the observed significant angular changes at individual joints on the trailing toe-obstacle clearance in the MCI group (black stick figure) compared to Control (**A**, grey stick figure) when the trailing toe was above an obstacle of 30% LL in height. The stick model was drawn using marker positions of a typical subject from each group. The segments with solid grey circles are joints of the reference limb. With the stance foot fixed to the ground, only one joint was rotated at a time according to the mean angular change reported in Tables 3, 5, while keeping the angles of the other joints fixed, and the segments of the stance limb and the segments of the swing limb distal to the current joint stationary. The MCI group showed decreased pelvic posterior tilt **(B)** and decreased ankle dorsiflexion in the swing limb **(C)**. As indicated by the black stick figure, **(B)** tended to increase the toe-obstacle clearance while **(C)** gave the opposite effect, leading to the observed normal trailing toe-obstacle clearance. Note that the stick figures were drawn based on the statistical results of the averaged values of both limbs reported in Tables 3, 5.

When the leading toe was above the obstacle, the MCI group showed significantly increased pelvic anterior tilt, hip abduction, and knee adduction in the swing limb (Tables 3, 5). When the trailing toe was above the obstacle, the MCI group showed significantly decreased ankle dorsiflexion and pelvic posterior tilt in the swing limb (Tables 4, 5). The observed significant angular changes at individual joints showed different effects on the leading and trailing toe-obstacle clearances in the MCI group when compared with the Control, some tending to increase the toe-obstacle clearance while others showed opposite effects (Figures 3, 4).

**Table 3:** Means (standard deviations) of the crossing angles of the pelvis relative to the global norm in patients with mild cognitive impairment (MCI) and healthy controls (Control) when the leading or trailing toe was above the obstacles of heights of 10, 20, and 30% of the subjects’ leg length (LL).

Variables	Group	Obstacle height (% LL)			Main effect
		10%	20%	30%	P_G_, P_H_
**Leading toe above obstacle**					
Upward (+)/downward (−) list	MCI	4.3 (3.6)	6.8 (3.9)	8.9 (4.6)	0.726, <0.001↑
	Control	3.6 (2.5)	6.7 (3.9)	11.2 (5.9)	
Ipsi (+)/contra (−) rotation	MCI	−2.6 (3.1)	−4.3 (5.3)	−4.9 (6.7)	0.518, 0.018↓
	Control	−1.7 (2.5)	−4.4 (4.6)	−6.5 (5.9)	
Anterior (+)/posterior (−) tilt	MCI	2.7 (4.6)	4.4 (5.3)	6.2 (5.6)	0.037*, <0.001↑
	Control	0.9 (3.2)	3.1 (4.6)	5.2 (4.3)	
**Trailing toe above obstacle**					
Upward (+)/downward (−) list	MCI	2.1 (1.8)	1.0 (2.6)	−0.5 (3.3)	0.627, <0.001↓
	Control	2.5 (0.8)	1.1 (2.2)	0.1 (2.9)	
Ipsi (+)/contra (−) rotation	MCI	−2.6 (3.1)	−4.3 (5.3)	−4.9 (6.7)	0.017*, 0.001↓
	Control	−5.6 (3.5)	−8.6 (6.0)	−12.8 (6.4)	
Anterior (+)/posterior (−) tilt	MCI	−2.7 (4.3)	−3.3 (5.2)	−6.1 (6.0)	0.042*, <0.001↓
	Control	−3.8 (3.4)	−4.7 (4.5)	−7.9 (5.6)	

*P*-values for main group and height effects are given as no interactions were found. P_G_, MCI vs. Control; P_H_, value of p for obstacle height; *indicates a significant group effect (P_G_ < 0.05); ↑ indicates a linearly increasing trend and ↓ indicates a linearly decreasing trend (P_H_ < 0.05). Upward list (+) indicates that the contralateral hip joint center is higher than the ipsilateral hip; Ipsi rotation (+) indicates that the ipsilateral hip joint center is anterior to the contralateral hip; Ipsi, ipsilateral, Contra, contralateral.

**Table 4:** Means (standard deviations) of the crossing angles of the hip, knee, and ankle joints of the leading swing limb and trailing stance limb in the patients with mild cognitive impairment (MCI) and healthy controls (Control) when the leading toe was above the obstacle of heights of 10, 20 and 30% of the subject’s leg length (LL).

Variables	Group	Obstacle height (% LL)			Main effect
		10%	20%	30%	P_G_, P_H_
**Leading swing limb**					
Hip					
Flexion (+)/extension (−)	MCI	56.9 (5.5)	65.9 (5.4)	71.5 (5.7)	0.465, <0.001↑
	Control	58.3 (7.6)	67.2 (7.4)	74.5 (7.0)	
Adduction (+)/abduction (−)	MCI	−0.2 (4.0)	−3.1 (5.0)	−4.5 (6.1)	0.042*, <0.001↑
	Control	1.9 (2.7)	−0.5 (4.1)	−2.7 (3.2)	
Knee					
Flexion (+)/extension (−)	MCI	92.7 (8.0)	108.1 (10.0)	117.0 (9.2)	0.835, <0.001↑
	Control	90.3 (11.2)	106.7 (11.8)	118.4 (11.1)	
Adduction (+)/ abduction (−)	MCI	−2.0 (10.4)	0.6 (11.7)	2.4 (14.0)	0.036*, 0.099
	Control	−11.4 (10.9)	−11.5 (13.6)	−10.7 (16.4)	
Ankle					
Dorsiflexion (+)/plantarflexion (−)	MCI	7.3 (3.9)	7.3 (3.6)	8.9 (4.7)	0.405, 0.002↑
	Control	9.5 (5.1)	8.9 (5.0)	10.0 (6.4)	
Adduction (+)/abduction (−)	MCI	−1.4 (3.3)	−2.1 (3.2)	−2.4 (3.6)	0.377, 0.023↑
	Control	0.0 (4.3)	−0.6 (4.2)	−0.9 (4.9)	
**Trailing stance limb**					
Hip					
Flexion (+)/extension (−)	MCI	0.9 (7.2)	2.0 (7.1)	1.9 (6.6)	0.435, 0.330
	Control	3.4 (3.9)	3.5 (4.2)	3.4 (4.7)	
Adduction (+)/abduction (−)	MCI	2.1 (4.4)	0.0 (4.1)	−2.6 (5.4)	0.316, <0.001↓
	Control	4.1 (2.1)	2.2 (3.0)	−1.9 (5.2)	
Knee					
Flexion (+)/extension (−)	MCI	7.7 (6.7)	8.4 (5.9)	8.5 (6.5)	0.878, 0.722
	Control	8.8 (6.3)	8.9 (6.4)	8.2 (6.6)	
Adduction (+)/abduction (−)	MCI	−1.1 (3.6)	−1.4 (2.9)	−1.5 (3.0)	0.627, 0.202
	Control	−1.7 (2.5)	−2.0 (2.9)	−2.1 (3.2)	
Ankle					
Dorsiflexion (+)/plantarflexion (−)	MCI	2.9 (2.4)	2.6 (2.8)	1.9 (2.9)	0.923, <0.001↓
	Control	3.7 (2.0)	2.9 (1.9)	1.1 (2.7)	
Adduction (+)/abduction (−)	MCI	−4.5 (1.8)	−4.6 (2.1)	−3.8 (2.5)	0.681, 0.050
	Control	−4.9 (2.3)	−5.1 (2.4)	−3.8 (2.0)	

*P*-values for main group and height effects are given as no interactions were found. P_G_, MCI vs. Control; P_H_, value of p for obstacle height; *indicates a significant group effect (P_G_ < 0.05); ↑ indicates a linearly increasing trend and ↓ indicates a linearly decreasing trend (P_H_ < 0.05).

**Table 5:** Means (standard deviations) of the crossing angles of the hip, knee, and ankle joints of the trailing swing limb and leading stance limb in the patients with mild cognitive impairment (MCI) and healthy controls (Control) when the trailing toe was above the obstacle of heights of 10, 20, and 30% of subjects’ leg length (LL).

Variables	Group	Obstacle height (% LL)			Main effect
		10%	20%	30%	P_G_, P_H_
**Trailing swing limb**					
Hip					
Flexion (+)/extension (−)	MCI	29.7 (5.2)	32.4 (5.9)	35.1 (7.3)	0.593, <0.001↑
	Control	26.5 (6.4)	31.8 (7.0)	35.0 (7.8)	
Adduction (+)/abduction (−)	MCI	−2.0 (2.5)	−1.9 (2.9)	−2.6 (2.7)	0.511, 0.077
	Control	−1.0 (3.6)	−1.2 (3.3)	−2.0 (3.4)	
Knee					
Flexion (+)/extension (−)	MCI	101.7 (11.0)	114.1 (12.1)	127.4 (12.7)	0.965, <0.001↑
	Control	98.7 (17.5)	114.9 (16.1)	129.0 (15.3)	
Adduction (+)/abduction (−)	MCI	−10.2 (7.2)	−8.7 (6.6)	−6.9 (7.1)	0.277, <0.001↑
	Control	−12.1 (7.3)	−12.2 (8.2)	−11.4 (7.5)	
Ankle					
Dorsiflexion (+)/plantarflexion (−)	MCI	−10.2 (9.4)	−4.5 (6.7)	1.9 (4.1)	0.038*, 0.028↑
	Control	−3.7 (10.5)	0.8 (6.1)	5.0 (7.8)	
Adduction	MCI	−2.1 (2.6)	−3.1 (2.6)	−2.8 (3.9)	0.328, 0.560
	Control	−0.6 (6.0)	−0.9 (7.0)	−0.3 (7.1)	
**Leading stance limb**					
Hip					
Flexion (+)/extension (−)	MCI	10.9 (6.8)	11.3 (7.5)	11.8 (6.8)	0.777, 0.185
	Control	12.2 (3.1)	11.4 (3.5)	12.3 (4.6)	
Adduction (+)/abduction (−)	MCI	5.9 (2.6)	4.2 (2.9)	1.3 (4.6)	0.730, <0.001↓
	Control	6.3 (2.6)	3.1 (5.5)	0.3 (5.3)	
Knee					
Flexion (+)/extension (−)	MCI	9.0 (7.7)	8.3 (7.7)	6.5 (7.1)	0.588, <0.001↓
	Control	8.7 (5.7)	6.5 (5.2)	4.4 (4.8)	
Adduction (+)/abduction (−)	MCI	0.1 (3.0)	−0.1 (3.3)	0.2 (3.3)	0.852, 0.123
	Control	−0.4 (2.5)	−0.2 (2.4)	0.1 (2.3)	
Ankle					
Dorsiflexion (+)/plantarflexion (−)	MCI	0.9 (2.9)	1.4 (2.9)	0.8 (2.7)	0.821, 0.058
	Control	1.5 (2.2)	1.0 (2.1)	−0.0 (2.3)	
Adduction (+)/abduction (−)	MCI	−2.4 (1.8)	−2.8 (1.5)	−1.8 (2.0)	0.868, 0.089
	Control	−2.7 (2.9)	−2.0 (4.3)	−1.6 (4.0)	

*P*-values for main group and height effects are given as no interactions were found. P_G_, MCI vs. Control; P_H_, *p*-value for obstacle height; *indicates a significant group effect (P_G_ < 0.05); ↑ indicates a linearly increasing trend and ↓ indicates a linearly decreasing trend (P_H_ < 0.05).

There were no interactions between the group and height factors for any of the variables. With increasing obstacle height, both MCI and Control linearly reduced their crossing speeds, but linearly increased their trailing toe-obstacle clearance (Table 2). When the leading toe was above the obstacle, both groups linearly increased the pelvic upward list, contralateral rotation and anterior tilt, hip flexion and abduction, knee flexion, ankle dorsiflexion and abduction, but linearly decreased the pelvic downward list of the swing limb, as well as linearly decreased the hip adduction and ankle dorsiflexion of the stance limb (Tables 3, 5). On the other hand, when the trailing toe was above the obstacle, both groups linearly decreased the pelvic upward list and knee abduction, but increased the pelvic contralateral rotation and posterior tilt, hip flexion, knee flexion, and ankle dorsiflexion of the swing limb, as well as linearly decreased the hip adduction and knee flexion of the stance limb (Tables 4, 5).

## Discussion

The current study aimed to identify the kinematic strategies of the pelvis-leg apparatus in patients with single-domain aMCI when crossing obstacles of three different heights. Compared to healthy controls, the patients were found to cross obstacles with increased pelvic anterior tilt, swing hip abduction and knee adduction during leading-limb crossing, and decreased pelvic posterior tilt, swing ankle dorsiflexion during trailing-limb crossing. Such a specific kinematic strategy enabled the MCI group to maintain the leading and trailing toe-obstacle clearances similar to those of their healthy peers for all tested obstacle heights. The current results suggest that regular monitoring of obstacle-crossing kinematics for reduced toe-obstacle clearance or any signs of changes in crossing strategy may be helpful for early detection of compromised ability to cross obstacles in patients with single-domain aMCI.

During leading-limb crossing, the specific strategy observed in the MCI group for normal leading toe-obstacle clearance involved primarily sagittal-plane kinematic changes in the pelvis and frontal-plane kinematic changes in the swing limb, namely increased pelvic anterior tilt, hip abduction, and knee adduction in the swing limb. While the increased pelvic anterior tilt would have decreased the leading toe-obstacle clearance (Figure 3) – an indication of increased risk of tripping as toe-obstacle contact is more likely to occur (Sparrow et al., 1996) – the changes at the hip and knee would have increased the leading toe-obstacle clearance. In other words, the potential unfavorable downward deviations of the end-point owing to the increased pelvic anterior tilt were compensated for by the increased hip abduction and knee adduction. Being proximal to the hip and knee, the pelvis had a greater effect on the position of the end-point and thus the toe-obstacle clearance (Chen et al., 2016; Wu et al., 2019b), requiring kinematic changes at two distal joints to provide compensation. A different compensatory mechanism was also observed during trailing-limb crossing. While the decreased pelvic posterior tilt would have increased the trailing toe-obstacle clearance (Figure 4), such potential favorable upward deviations of the end-point were neutralized by the opposite effects from the decrease in the swing ankle dorsiflexion, resulting in the observed normal toe-obstacle clearance. The altered pelvic motions in MCI during leading-limb crossing not only had potential negative effects on the end-point, but may also affect the rotation of the trunk and thus the dynamic balance control. This is critical as the neuromechanical challenges for balance control are already greater during leading-limb crossing than trailing-limb crossing. During leading-limb crossing the body is moving away from the trailing stance limb while it is moving towards the leading stance limb during trailing-limb crossing (Wu et al., 2020). Further studies on the motion of the upper body will be needed to gain more insight into the effects of the observed kinematic strategy on the balance control in patients with aMCI. The current results suggest that patients with aMCI can be distinguished from their healthy peers mainly by the increased pelvic anterior tilt during leading-limb crossing and decreased pelvic posterior tilt during trailing-limb crossing, of which the effects on toe-obstacle clearance were compensated for by swing limb kinematic changes at the hip and knee and at the ankle, respectively. Such kinematic features in MCI were not affected by obstacle heights up to 30% of the leg length, as indicated by the independence between the height and group effects (Tables 2–5).

While the precise mechanism underlying the observed kinematic strategy for obstacle-crossing in the current patients with aMCI could not be identified with the current results, a plausible possibility of the connections between the kinematic changes and impaired cognitive function is related to the degeneration of the hippocampus that leads to memory deficits and impaired cognitive functions in MCI (Scheff et al., 2006). Memory involves the acquisition and maintenance of relevant sensory stimuli used to guide movements (Jonides et al., 2008), which provide the ability to cross obstacles without directly looking at the lower limbs and the obstacle (Patla and Vickers, 1997; Mohagheghi et al., 2004). The hippocampus thus plays an important role in the ability of working memory regarding the environment with an obstacle and the ability to reassign spatial orientation and navigation precisely to and from the obstacle before and during crossing (Wilkinson and Sherk, 2005; Whishaw et al., 2009). The hippocampus is involved not only in the formation and storage of long-term memories but also in the integration of visual, vestibular, and proprioceptive sensory and contextual information into spatial maps (Nutt et al., 1993; Scherder et al., 2007). Degeneration of the hippocampus thus has a direct impact on the performance of obstacle-crossing when the ability of spatial orientation and navigation is affected (Buzsáki and Moser, 2013; Eichenbaum and Cohen, 2014).

Apart from the degeneration of the hippocampus, the prefrontal cortex may also play a role in the formation of the kinematic strategy for obstacle-crossing in MCI, as both brain regions have been shown to have a positive relationship, especially during a spatial working memory task (Sigurdsson and Duvarci, 2016). The prefrontal cortex is a key structure for executive functions (Funahashi and Andreau, 2013) and is the main brain region shared by the control of cognitive and gait function under the neural control mechanisms (Cohen et al., 2016). Executive functions refer to a set of cognitive processes, including mental flexibility, planning, decision-making, and cognitive control of behavior (Shallice and Burgess, 1991; Miyake et al., 2000). Obstacle-crossing requires not only the ability of motor planning (Clark et al., 2014), but also an adequate allocation of cognitive resources, including memory (Patla and Vickers, 1997; Mohagheghi et al., 2004), attention and executive function (Clark et al., 2014; Clark, 2015). Both the hippocampus and prefrontal cortex are responsible for memory, attention, and executive functions and are associated with the changes in temporal–spatial gait parameters of patients with MCI, and with avoiding obstacles (Malouin et al., 2003; Allali et al., 2016; McGough et al., 2018; Beauchet et al., 2020). Individuals with aMCI and additional executive dysfunction also show a greater risk of a misstep from choosing the incorrect motor responses during walking in a complex obstructed walkway that requires some decision-making, mental flexibility, or cognitive control of behavior (Persad et al., 2008). Although the current patients were impaired with single-domain aMCI, they showed significantly lower scores in global cognition, executive function, and attention compared to healthy controls (Table 1). It appears that the observed specific kinematic strategies for obstacle-crossing in the current patients were a result of both compromised ability of spatial orientation and navigation associated with the degeneration of the hippocampus, and the compromised executive function associated with the prefrontal cortex. Further comparative studies on multi-domain aMCI may be needed to identify the specific effects of impairments of domains such as executive functions and attention on the kinematics of obstacle-crossing.

The current study was the first to identify the kinematic strategies used by patients with amnestic MCI during obstacle-crossing. Further studies on patients with multiple-domain aMCI and non-amnestic MCI would be needed to determine whether kinematic strategies for obstacle-crossing would change with different cognitive impairment subtypes or with the progression of the disease. Individuals with cognitive deficits display a decreased ability to estimate balance hazards when navigating, particularly under increased cognitive challenges (Pieruccini-Faria et al., 2019). Further studies may also examine the effects of the neurological pathway mediating both cognitive and motor functions *via* dual-tasking on the kinematic strategies in older people with MCI or populations with increased fall risks. Further studies on the kinematic strategies under cognitive/motor dual-task conditions will be needed to better identify the roles of degraded cognitive function in the observed postural adjustments during obstacle-crossing. Understanding the similarities or differences in the control strategies and the resulting joint mechanics between normal and patient groups would be useful for developing improved fall-prevention strategies and for making better use of the obstacle-crossing task in rehabilitation programs.

## Conclusion

The current study identified the kinematic changes in the pelvis-leg apparatus in patients with single-domain aMCI during obstacle-crossing as compared to healthy controls. The patients were able to maintain normal leading and trailing toe-obstacle clearances for all tested obstacle heights with a specific kinematic strategy, namely increased pelvic anterior tilt, swing hip abduction, and knee adduction during leading-limb crossing, and decreased pelvic posterior tilt and swing ankle dorsiflexion during trailing-limb crossing. The current results suggest that regular monitoring of obstacle-crossing kinematics for reduced toe-obstacle clearance or any signs of changes in crossing strategy may be helpful for early detection of compromised ability to cross obstacles in patients with single-domain aMCI. Further studies using a motor/cognitive dual-task approach in patients with multiple-domain MCI may be needed for a complete picture of the functional adaptations in such patient groups.

## Data availability statement

The original contributions presented in the study are included in the article/supplementary material, further inquiries can be directed to the corresponding authors.

## Ethics statement

The studies involving human participants were reviewed and approved by Taipei Medical University Joint Institutional Review Board. The patients/participants provided their written informed consent to participate in this study.

## Author contributions

S-HL: conceptualization, data curation, resources, formal analysis, and writing—original draft. Y-CK, K-WW, and Y-LT: data curation, resources, and writing—original draft. H-YL: data curation, formal analysis, and writing—original draft. H-HC: conceptualization and writing—review and editing. T-WL: conceptualization, supervision, writing—original draft, and writing—review and editing. All authors contributed to the article and approved the submitted version.

## Funding

This work was supported by grants from the Taipei Medical University-Shuang Ho Hospital (108TMU-SHH-05) and Ministry of Science and Technology, Taiwan (MOST 108-2314-B-038-057 and 109-2628-B-038-010).

## Conflict of interest

The authors declare that the research was conducted in the absence of any commercial or financial relationships that could be construed as a potential conflict of interest.

## Publisher’s note

All claims expressed in this article are solely those of the authors and do not necessarily represent those of their affiliated organizations, or those of the publisher, the editors and the reviewers. Any product that may be evaluated in this article, or claim that may be made by its manufacturer, is not guaranteed or endorsed by the publisher.
